# Global multi-hazard risk assessment in a changing climate

**DOI:** 10.1038/s41598-024-55775-2

**Published:** 2024-03-11

**Authors:** Zélie Stalhandske, Carmen B. Steinmann, Simona Meiler, Inga J. Sauer, Thomas Vogt, David N. Bresch, Chahan M. Kropf

**Affiliations:** 1 Institute for Environmental Decisions, ETH Zurich, 8092 Zurich, Switzerland; 2 Federal Office of Meteorology and Climatology MeteoSwiss, Zurich-Airport, 8058 Zurich, Switzerland; 3 Potsdam Institute for Climate Impact Research, Potsdam, 14473 Germany

**Keywords:** Natural hazards, Climate sciences

## Abstract

Natural hazards pose significant risks to people and assets in many regions of the world. Quantifying associated risks is crucial for many applications such as adaptation option appraisal and insurance pricing. However, traditional risk assessment approaches have focused on the impacts of single hazards, ignoring the effects of multi-hazard risks and potentially leading to underestimations or overestimations of risks. In this work, we present a framework for modelling multi-hazard risks globally in a consistent way, considering hazards, exposures, vulnerabilities, and assumptions on recovery. We illustrate the approach using river floods and tropical cyclones impacting people and physical assets on a global scale in a changing climate. To ensure physical consistency, we combine single hazard models that were driven by the same climate model realizations. Our results show that incorporating common physical drivers and recovery considerably alters the multi-hazard risk. We finally demonstrate how our framework can accommodate more than two hazards and integrate diverse assumptions about recovery processes based on a national case study. This framework is implemented in the open-source climate risk assessment platform CLIMADA and can be applied to various hazards and exposures, providing a more comprehensive approach to risk management than conventional methods.

## Introduction

Climate change is affecting natural and socio-economic systems in all parts of the world. In this context, the Intergovernmental Panel on Climate Change (IPCC) has defined climate risks as “arising from the dynamic interactions between climate-related hazards and the exposure and vulnerability of affected human and ecological systems”^1^. This definition has been taken up by many studies that typically focus on a single hazard affecting an exposure^2^. However, since multiple hazards can challenge these systems, focusing on a single hazard can result in an incomplete assessment of the risk. Combining risks from multiple single hazards by assuming them to be independent is a first step towards a more comprehensive risk assessment. This type of risk assessment is defined by the European Commission as multi-layer single-hazard risk assessment^3^. This allows to compute a shared exceedance probability of losses using individual risk estimates^4^. However, this approach neglects the effects of temporal sequence and co-occurrence of events on risk. Therefore, there have been international calls for more attention to multi-hazards and multi-risks^5^ and for harmonization of related definitions^3,6,7^.

The European Commission defines multi-hazard risk as the assessment of the interactions between single hazards, in contrast to assessing them independently from one another^3^. These interactions are classified into five groups based on Gill and Malamud’s work in 2014 and 2016. Hazards can trigger a subsequent event (1), increase (2) or decrease (3) the probability of another hazard; they can coincide (4), or catalyse/impede (5) one another^8,9^. Based on these hazard interactions, the resulting risk assessment is considered as multi-hazard risk. Furthermore, including dynamic vulnerabilities results in multi-risk assessments.

In parallel, the concept of compound events has emerged in recent years in weather and climate science. In that context, compound events are defined as the combination of multiple drivers and/or hazards that contribute to societal or environmental risk^10^. The term compound has typically been applied to describe events co-occurring over meteorological timescales (hours to weeks), and to express the resulting intensification of the negative consequences^11^. Complementary, Hillier et al. propose the definition of compound risks to include risks that compound on extended time-frames which are more relevant for decision making; and whose compounding can also lead to decreased risks^11^. This definition recognizes that private and public organizations face an increasing global exposure to natural hazards and that methods that account for atmospheric connections between hazards (tele-connections) are necessary to manage and mitigate risks. If we account for these connections at the relevant time and spatial scale for a given decision-making process, we can provide a more accurate evaluation of risks, preventing overestimation and underestimation, which can both be problematic^11^.

When transitioning from analyzing single hazards to multiple hazards, it is necessary to establish a common timescale to consider how these hazards interact^12^. As one progresses to examining the impacts and risks of these hazards, a choice must be made regarding how to model the exposures and vulnerabilities on the defined timescale. Merely assuming static exposures and vulnerabilities can result in inconsistencies^13^. For example, consider a house that is destroyed by both a tornado and a wildfire in the same week. If this house is counted as destroyed twice, it would be inaccurate as it cannot be reconstructed within a week. In other words, when assessing compound risks of multiple hazards, it is essential to consider the assumptions made about the recovery of the exposures and changes in their vulnerabilities^3,14^.

In this study, we provide a framework for assessing multi-hazard risks under different climate conditions. We build upon the compound risk definition provided by Hillier et al.^11^ to investigate spatially compounding risk occurring in the same time window (e.g., a day, a season, or a year) on a global scale; and spatio-temporally compounding risk occurring at the same location in the same time window. We focus on global river floods (RF) and tropical cyclones (TC), the two hazards currently affecting the largest number of people and causing the highest damage to physical assets^15^. We use TC and RF footprints from the Inter-Sectoral Impact Model Intercomparison Project ISIMIP models^16^ that take the same set of Global Climate Models (GCMs) as input. This basis of common climatic inputs allows combining hazard (RF and TC) realisations that share physical drivers, even if they were modelled independently from one another. In order to showcase their effects on different exposure types, we assess the impacts of both hazards on population and economic assets. We chose to report annual risk metrics, as they are of high relevance for decision-making^17^. With the IPCC showing that human activities are responsible for a global warming of about $1.1\;^{\circ }$C since pre-industrial levels (1850–1900)^18^, we choose model simulations based on GCM runs at $1\;^{\circ }$C warming level as representation of today’s world. Moreover, with climate change exacerbating the frequency and intensity of natural hazards^18^, we additionally assess the risk in a $2\;^{\circ }$C world. We extend our analysis to include heat stress (HS) as an additional hazard in a case study focused on Vietnam, examining impacts solely on the population. In the context of this case study, we additionally test different assumptions of recovery times for asset damages from TC and RF to illustrate how these could be integrated in this framework. We finally discuss the requirements for advancing towards a complete multi-risk assessment. All methods are implemented in the open-source climate-risk assessment platform CLIMADA^19^, which allows the analysis of probabilistic event sets under present and future climatic conditions. A multitude of hazards are represented in CLIMADA and can be considered in future work on multi-hazard risk. This study is the first to our knowledge to assess multi-hazard risks on an event basis at a global scale in a consistent fashion across GCMs.

## Results

To assess spatially compounding and spatio-temporally compounding multi-hazard impacts, we combine single-hazard impacts with a four-step process. The single-hazard sets contain individual events. The impact of each event is calculated by combining the three components hazard, exposure and vulnerability. The vulnerability of the given exposure is described by an impact function which constitutes the relationship of the respective hazard intensity and the caused impact^19^. Event impacts are computed separately for each exposure type at every exposure location (see “Methods” for more details on the single hazard risk calculation). Second, we define a time period of interest, e.g. one calendar year *y*. In order to compute annual impacts for one hazard we sum all event impacts of the respective hazard occurring within one year at each exposure location. Here one has to make an assumption about the recovery of the exposures between multiple events during the time period. The simplest case assumes full recovery every year which can be modelled by capping the annual impact at each location at the total value of the exposure at said location (please see “Methods” for different recovery times). When working with GCMs, each year *y* has a realisation driven by each GCM, resulting in the annual impact $I^{y,g}_{h,k}$ for hazard *h*, GCM model *g* in year *y* at location *k* given by

1 $$\begin{aligned} I^{y,g}_{h,k} = \text {min}\left( E_{k}, \sum _{\varepsilon = 1}^{L^{y,g}_h} I_{\varepsilon , h, k}\right) \end{aligned}$$

where $E_k$ is the value of the exposure at location *k*, $L^{y,g}_h$ the number of events $\varepsilon$ in year *y* for GCM *g* for hazard *h*, and $I_{\epsilon , h, k}$ the corresponding impact per event at location *k*. These single-hazard impacts are then added among hazards for the respective GCM year; and capped again. The spatially compounding multi-hazard global annual impact $S^{y,g}$ in year *y* for GCM *g* is

2 $$\begin{aligned} S^{y,g} = \sum _k \text {min}\left( E_{k}, \sum _{h} I^{y,g}_{h, k}\right) . \end{aligned}$$

By combining single-hazard years driven by the same year of the same GCM, common physical drivers can be maintained when adding impacts. Capping impacts at the exposure value at each point corresponds to assuming that the maximum compounded multi-hazard impact per year cannot exceed the exposure value (i.e., no recovery within a year). Computing these impacts for each year *y* independently assumes full recovery at the end of each calendar year. Longer recovery times can be accounted for by transferring damages from one year to another as shown for impacts on assets in the national case study below and as described in the methods. In the last step, the spatio-temporally compounding multi-hazard global annual impact $T^{y,g}_{h^n}$ for GCM *g* is obtained by summing the impact of all exposure locations *k* affected by events of the *n* desired hazards $h^{n}$ in the respective year *y*:

3 $$\begin{aligned} T^{y,g}_{h^n}= \sum _k {\left\{ \begin{array}{ll} \text {min}\left( E_{k}, \sum _{j=1}^n I^{y,g}_{h_j,k}\right) , &{} \text {if } I^{y,g}_{h_j,k} \ge 0 \text { for all } j \\ 0, &{} \text {otherwise} \end{array}\right. }. \end{aligned}$$

When studying three or more hazards, each grouping of *n* hazards $h^n$ would result in a spatio-temporally compounding multi-hazard global annual impact (see results in “Case study on national level”). Risk is calculated by multiplying the severity of events (here impacts Eqs. (1)–(3) with their probability of occurrence in the given time period.

In this work, we analyze 5000 years of spatially compounding and spatio-temporally compounding multi-hazard impacts for the global ISIMIP hazards TC and RF affecting the exposures physical assets and affected population. While the impact functions for assets are regional sigmoid curves, people are considered to be affected based on a threshold of 1 m flood depth (for RF) and 33 m/s wind speed (for TC) (See SI section 5.1). Affected could mean, for example, displaced, injured or experiencing a loss of livelihood. Capping impacts at the exposure value at each point corresponds to assuming that each asset can only be damaged fully once each year or that each person can only be affected once. Finally, this also assumes that both people and assets fully recover at the end of each calendar year. In order to study impacts and risks at different warming levels, we make use of the year $y_{w,g}$ in which a certain GCM *g* reaches a given warming level *w*. We extract 10 years before and after $y_{w,g}$ for the warming levels $1\;^{\circ }$C, $2\;^{\circ }$C and each of the 4 GCMs *g*. This results in a 21 years time window ($U_{w,h,g}$) per warming level *w*, hazard *h* and GCM *g*. Within each year the ISIMIP hazard sets contain one RF and several TC events. We then compute the spatially compounding (c.f., Eq. (2) and the spatio-temporally compounding multi-hazard global annual impact for the two hazards (c.f., Eq. 3)

4 $$\begin{aligned} T^{y,g}_{\text {TC}, \text {RF}}= \sum _k {\left\{ \begin{array}{ll} \text {min}\left( E_{k}, I^{y,g}_{\text {TC},k} + I^{y,g}_{\text {RF},k}\right) , &{} \text {if } I^{y,g}_{\text {TC},k} \ge 0 \text { and } I^{y,g}_{\text {RF},k} \ge 0 \\ 0, &{} \text {otherwise} \end{array}\right. }, \end{aligned}$$

where for clarity of notation we omitted the subscripts of $y_{w,g}$. Subsequently, we disregard the sequence of years within GCMs and use all years $U_{w}$ belonging to one warming level *w* for risk computations. Each spatially compounding or spatio-temporally compounding multi-hazard global annual impact has a probability $P=1/U_w$ to occur in year *y*, and the resulting risks are then $R_S^{y,g,w} = S^{y,g}\cdot 1/U_w$ and $R_T^{y,g,w} = T^{y,g}\cdot 1/U_w$, respectively. We note here that although the TC hazard comprises only wind, the impact functions are calibrated based on TC damages from all associated sub-hazards, including flooding^20^. Conversely, the RF model does not account for flooding caused specifically by TCs. Hence, in this context, TC and RF are treated as distinct hazards, ensuring that damages are not counted twice.

### Impact & risk maps

**Figure 1:**
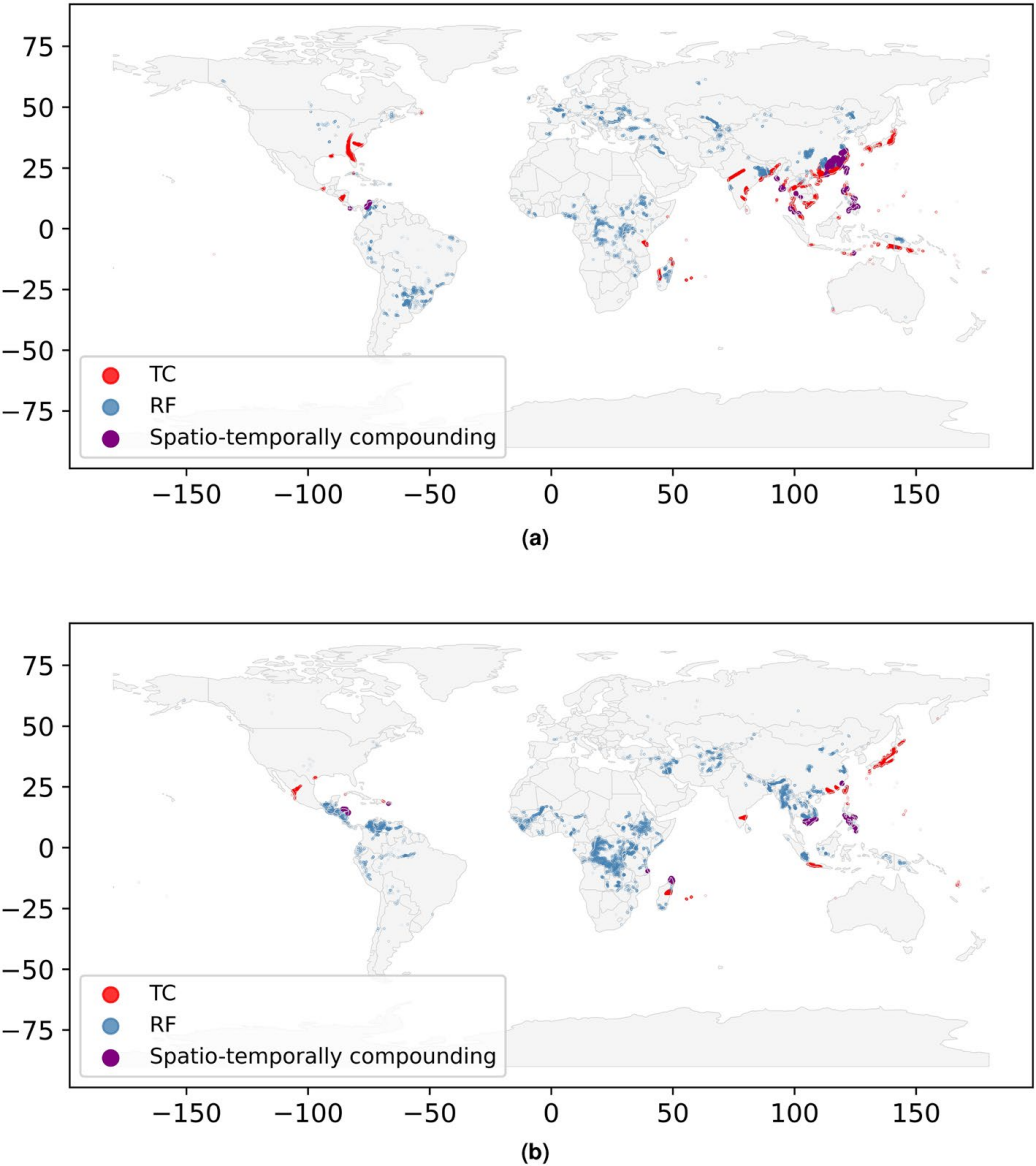
Illustration of the spatio-temporally compounding impact of TC and RF. Each exposure point is either impacted by TC (red) only, RF (blue) only, both (purple), or neither (grey). The impact is shown for the warming level $1\;^{\circ }$C for (**a**) assets and (**b**) population for a given example year chosen to best illustrate the compounding distribution heterogeneity. Note that the year for (**a**) is different than for (**b**). The maps were generated using CLIMADA V4.0.1 (https://zenodo.org/records/8383171).

With the defined spatio-temporally compounding impacts, we study the worldwide distribution of annual co-occurrence of RF and TC, as shown for example years $y_{1}$ and $y_{2}$ in Fig. 1. These years are chosen to be years were the spatio-temporally compounding impacts reach the value of a one in a hundred year impact (100-year impact) for the warming level $1\;^{\circ }$C. This means that on average every hundred years, we would expect this value of exposures affected by both hazards to be reached at that warming level. In the case of assets, this corresponds to the GCM HADGEM2-ES for the year 2012 under RCP6.0. In the case of population this corresponds to the GCM GFDL-ESM2M for the year 2008 under RCP2.6. The distribution is typically heterogeneous, with only few regions worldwide impacted by both TC and RF in these years ($I_{RF,k}>0$, $I_{TC,k}>0$), while most impacted regions experience either RF ($I_{RF,k}>0$, $I_{TC,k}=0$) or TC ($I_{RF,k}=0$, $I_{TC,k}>0$). This is both the case for assets (in a), where compounding takes place mostly in East Asia, Central America, and East Africa, and for population (in b), where compounding takes place mostly in East Asia and Central America.

Beyond individual years, one can also study common risk metrics such as the average annual impact (AAI), which is often used as a proxy for risk-based insurance premiums. The average annual impact is obtained by averaging the annual impact over all years corresponding to a given warming level. The AAI can be studied for different geographical scales, for example at each exposure point, by country or globally.

**Figure 2:**
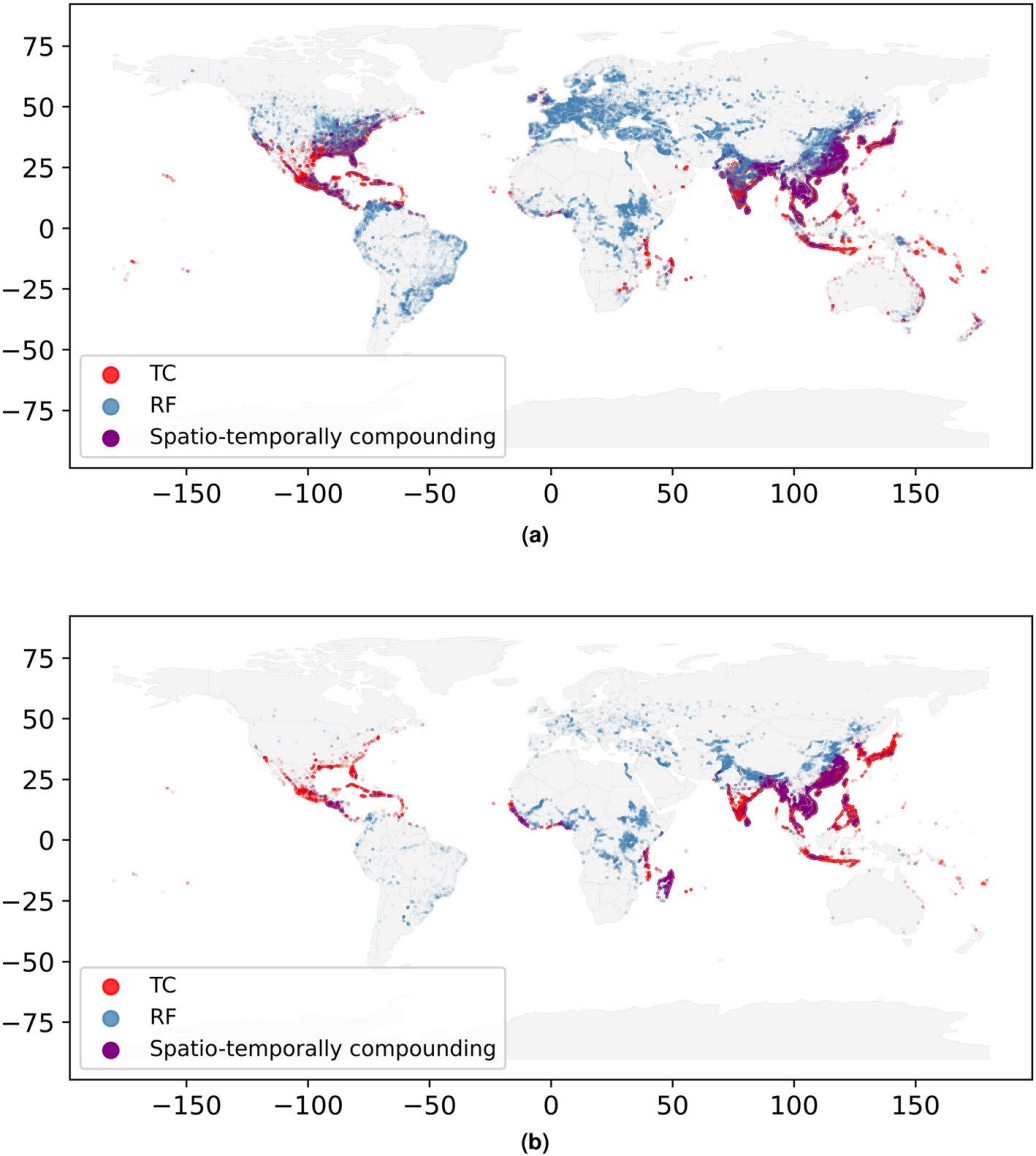
Exposure points affected by TC, RF and their compound on average per year at a $1\;^{\circ }$C warming level. Panel (**a**) shows exposure points with at least 1000 dollar damage, and panel (**b**) describes exposure points with at least 100 people affected on average per year. The maps were generated using CLIMADA V4.0.1 (https://zenodo.org/records/8383171).

Figure 2 shows exposure points that exceed 1000 dollars (in a) and 100 people affected (in b) in AAI. When examining the risk of the two hazards on population, we observe that fewer exposure points are affected. This can be attributed to the definition of vulnerability, were we consider 100% impact above the defined thresholds and no impact below. The spatio-temporally compounding scatter points in these maps highlight geographical areas for which adopting a multi-hazard risk or multi-risk framework is most relevant for the two studied hazards. Indeed, the exposures highlighted in purple are commonly affected by spatio-temporally compounding hazards. Recovery, as well as multi-hazard vulnerability may therefore be particularly important for those.

### Impact return period curves of spatially compounding risk

**Figure 3:**
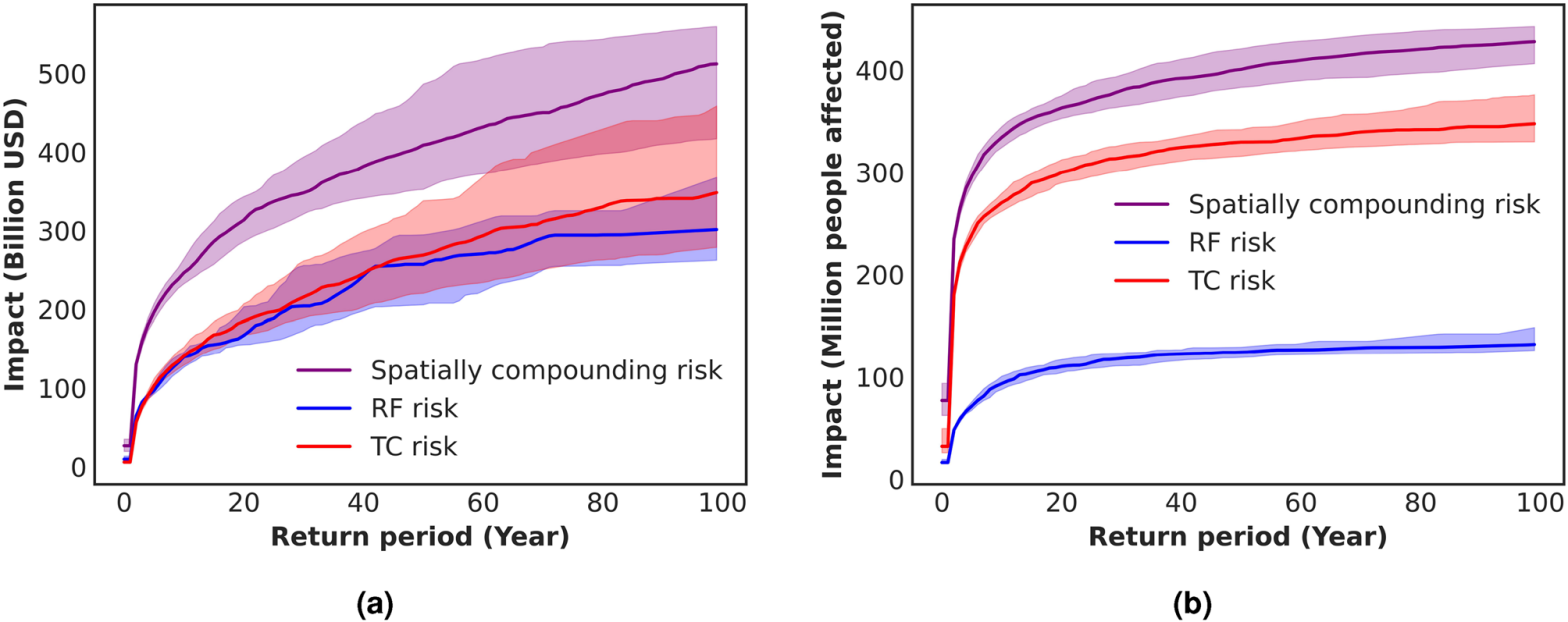
Impact return period curves for RF and TC at $1\;^{\circ }$C, illustrating the median and 90th percentile confidence intervals from 1000 samples of 500 years. Panel (**a**) represents assets, while panel (**b**) shows the impact on population.

An impact return period curve represents an additional valuable risk metric, which describes the probabilistic cumulative distribution of impacts. From this curve, one can for example read the 100-year impact, which is a useful metric that provides a standardized way of comparing the potential impact of natural hazards for a defined geographical area. It helps to guide risk management and mitigation strategies, and is used as a benchmark for determining insurance premiums and other financial policies related to natural hazards. Fig. 3 displays the impact return period curve for up to 100 years of RF, TC, as well as their spatially compounding risk curve at $1\;^{\circ }$C of global warming. In the case of assets, both hazards result in similar risk curves, while for population the TC risk is higher than for RF. Additionally, the spread between samples is larger for assets. These differences between population and assets can again be explained by the difference in vulnerability definition. We recall that population vulnerabilities are defined based on a threshold above which people are considered affected, while assets vulnerabilities are defined as regional sigmoid functions.

**Figure 4:**
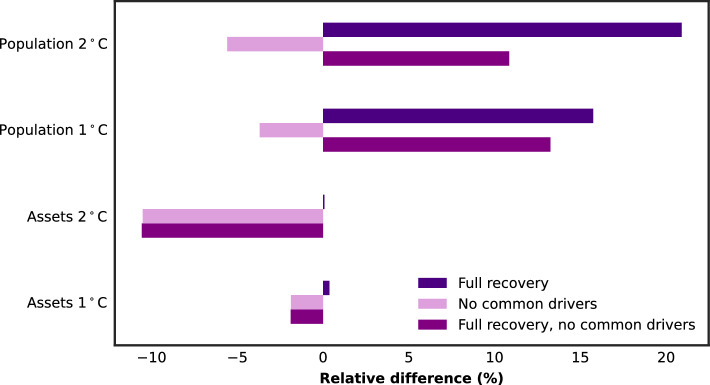
Relative difference in the average 100-year impact, if common physical drivers are not considered or/and if impacts are not caped at the exposure value (assuming full recovery between single events).

Figure 4 shows the relative difference to our estimates for the global 100-year impact of the spatially compounding risk, when neglecting the common physical drivers or/and the recovery. Common physical drivers are considered by combining single-hazard years driven by the same GCM (see Eq. (2)). We can also combine years randomly, which results in ignoring these common drivers. Recovery at the end of each year is integrated in our approach by capping the maximal impact per location *k* at the exposure value $E_{k}$ (see Eq. (2)). By neglecting this condition, we can also assume full recovery in between events. For population, the relative difference shows that the impact is larger when assuming full recovery between events. However, this assumption makes almost no difference for assets. This can be explained by the fact that assets are almost never entirely destroyed, while for population the vulnerability is binary. Neglecting common physical drivers leads to lower global impacts, especially at $2\;^{\circ }$C of warming, indicating that the role of common physical drivers might be increasing with higher temperatures. Considering both physical drivers and recovery, our methods results overall in a lower risk for population, and a higher risk for assets compared to neglecting physical drivers and recovery. In the case of spatio-temporally compounding risk (See SI section 2), combining years based on the same GCMs results in lower impacts at the same return periods compared to combining random years. This indicates that common drivers may decrease the occurrence of TC and RF at the same exposure points, emphasizing that the effect of those may differ for local multi-hazard risk assessments.

### Effect of climate change

**Figure 5:**
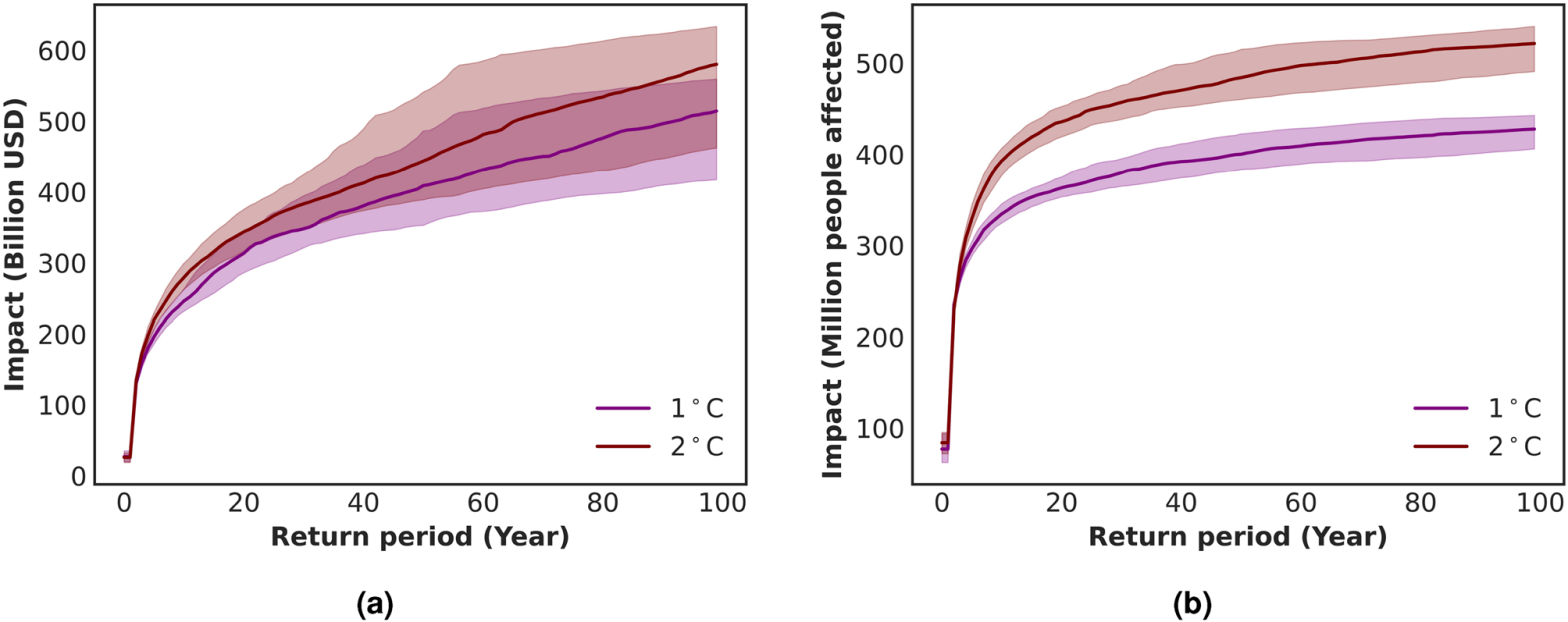
Impact return period curves for RF and TC at $1\;^{\circ }$C and $2\;^{\circ }$C, illustrating the median and 90th percentile confidence intervals from 1000 samples of 500 years. Changes in exposure are not considered in this analysis. Panel (**a**) represents assets, while panel (**b**) shows the impact on population.

In Fig. 5, we compare the spatially compounding risk curves for the present level of global warming ($1\;^{\circ }$C), from Fig. 3, with risk curves for further warming ($2\;^{\circ }$C). Only the change in climate is considered here, and we do not project socio-economic development. When comparing these two warming levels, the mean 100-year impact increases by 13% for assets (Fig. 5a) and by 22% for affected population (Fig. 5b) in a $2\;^{\circ }$C world. Moreover, Table 1 shows the contribution of the single hazards to this increase in combined impacts for the two studied exposures (see SI section 1 for the impact return period curves of the single hazard risks). It illustrates that the contribution of RF are more pronounced, in particular regarding increases in affected population under climate change. The increase in TC risks is more clearly observed for population, once more due to the definition of vulnerabilities. Table 1 additionally shows the relative change of the spatio-temporally compounding risks. Both the AAI and 100-year impact increase more than for the spatially compounding risks, and in the case of assets, more than for the single-hazards (See SI section 3 for the risk curves).

**Table 1:** Relative changes in impacts between 1 and $2\;^{\circ }$C in global warming for the two risk metrics 100-year impacts and AAI affecting the two exposures assets and population.

Risk metric		Assets	Population
100-year impact	Spatio-temporally compounding	28%	24%
	Spatially compounding	13%	22%
	Single-hazards	TC 10% | RF 11%	TC 20% | RF 28%
AAI	Spatio-temporally compounding	29%	28%
	Spatially compounding	11%	20%
	Single-hazards	TC 2% | RF 12%	TC 15% | RF 21%

### Case study on national level

In this section, we present a case study to illustrate a national assessment, and how a third hazard can be included. We choose Vietnam as a nation with a population that is particularly affected by compounding TC and RF risk (Fig. 2). We additionally consider heat stress (HS) as a hazard and its impact on the population. We define that population is affected at $33\;^{\circ }$C average daily wet bulb globe temperature (WBGT). The International Labor Organisation defines for instance that workers start facing a high risk of heat stress when the WBGT reaches about $33\;^{\circ }$C^21^. A daily mean WBGT of $33\;^{\circ }$C constitutes a quite extreme event that occurs with a frequency comparable to that of TCs or RFs, ensuring comparability in the assessment of different hazards.

**Figure 6:**
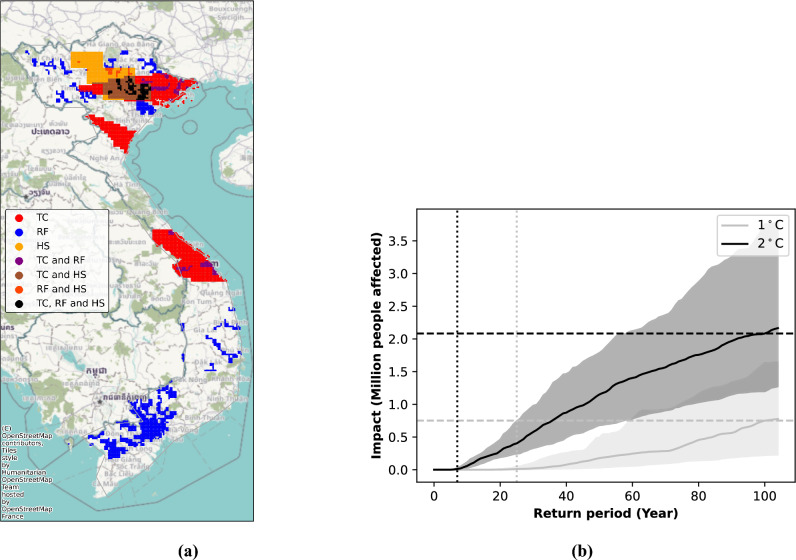
Illustration of a national case study for Vietnam, considering the impact of TC, RF, and HS. Panel (**a**) shows population exposure points affected by TC, RF, and HS for a spatio-temporally compounding 100-year event at $1\;^{\circ }$C global warming. Panel (**b**) illustrates the impact return period curve for people affected within the same year by these three hazards at $1\;^{\circ }$C and $2\;^{\circ }$C global warming. The map was generated using CLIMADA V4.0.1 (https://zenodo.org/records/8383171).

Figure 6a illustrates an event with an impact expected to occur statistically once in 100 years at $1\;^{\circ }$C of global warming. The 100-year impact at $1\;^{\circ }$C would affect around 0.7 million people, while at $2\;^{\circ }$C, the number of affected people rises to about 2 million (see dashed lines Fig. 6b). Moreover, the dotted vertical lines illustrate the return period at which at least 10,000 people are affected by all three hazards in 1 year at each level of warming. This co-occurrence affects people roughly every 25 years at $1\;^{\circ }$C, while the frequency increases significantly at $2\;^{\circ }$C, with such events occurring as often as every 8 years. During the event depicted on Fig. 6a, the agglomeration of Hanoi is affected during that year by all three hazards, leading to a high number of people being affected. Other examples of 100-year events are illustrated in the SI section 4.1 at both $1\;^{\circ }$C and at $2\;^{\circ }$C.

**Figure 7:**
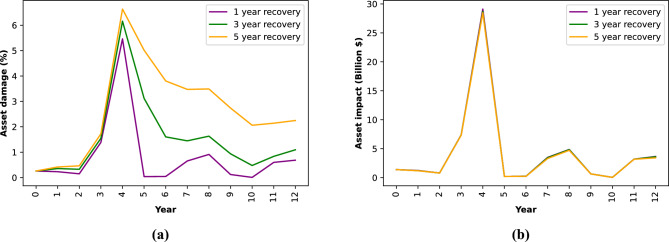
Time series of assets damaged and new impacts in Vietnam for the HadGEM2-ES RCP2.6 2006–2018 GCM run based on different recovery assumption for combined TC and RF. Panel (**a**) presents the percentage of assets value damaged. Panel (**b**) shows the new asset impacts.

In Fig. 7, we show a visual representation of how varying recovery assumptions influence the total impact in Vietnam on assets from both TC and RF for a time series of a single model at $1\;^{\circ }$C (See SI Section 4.2 for the recovery at one exposure point). Figure 7a depicts the proportion of assets that remain damaged in a particular year (residual damage), factoring in the cumulative effects of damage from previous years. This can be interpreted for example as infrastructure that has not been completely restored to functionality due to previous events. Conversely, Fig. 7b illustrates the new impact occurring within each year, which would correspond to the cost of assets that need to be rebuilt. While recovery assumptions significantly affect the former analysis, they have minimal impact on the latter, as it is uncommon for all assets in a grid cell to be completely destroyed. Note that this is likely sensitive to the resolution of the exposure layer, which here at $4 \times 4$ km does not resolve individual buildings. Note also that in this illustrative study, we kept the vulnerability constant during the recovery.

## Discussion

In this study, we develop a novel framework to assess multi-hazard risk for present and future climate conditions in a globally consistent way. This framework differs radically from the most naive approach consisting in assessing risks for each hazard individually and deciding on adaptation or risk transfer options for each hazard separately. The latter occurs for instance when developing separate insurance products for two independent hazards. This then results in total reserves designed to buffer the sum of both risks (e.g., of the 100-year impacts) which are likely to overestimate the actual need. Indeed, it is highly unlikely that both 100-year impacts occur in the same year. For independent hazards, the estimation of the 100-year spatially compounding impacts can be improved by computing the shared exceedance probability of losses by considering the impacts of all events of both hazards, ignoring which one caused the impact^4^. However, this approach is unsuited to assess risks when the hazards are strongly correlated, for instance, due to common physical drivers. Our framework addresses this issue by accounting for these common physical drivers consistently and across climate projections. Interestingly, in the example of TCs and RFs studied in this work, including common physical drivers in the risk assessment leads to higher global risk compared to considering the hazards to be independent.

Furthermore, our framework allows to include assumptions on recovery. Indeed, the use of impact time series allows the inclusion of recovery of the vulnerability and exposure over time. In the global RF and TC example presented here, we consider the limiting case that vulnerability remains unchanged and that exposure has fully recovered after each calendar year, but does not recover within the year. This assumption is reasonable for the affected population, such as those who have been displaced, and for low-impact on physical assets, such as shattered windows that can be replaced quickly. However, it may not be optimal for stronger impacts, which may lead to the reconstruction of collapsed buildings, which likely take longer and result in increased vulnerability during the rebuilding process^22^. These assumptions lead to lower overall risks when compared to the case when no recovery was included, especially in the case of population. Note that this is mainly because we do not include recovery within a given year (thus, the maximum impact for all RF and TC events combined within a given year is at each location maximally equal to the value of the exposure at this location).

Including recovery information as well as combining multiple hazards with common physical drivers consistently gives rise to complex interactions with non-trivial consequences for the overall risk assessment. In our global analysis of TC and RF, when considering both physical drivers and recovery, we obtain lower overall risks for population, and higher overall risks for assets compared to neglecting both factors.

While the effect of recovery generally lowers the impacts in our global analysis, we additionally test including different recovery times for a series of consecutive years of asset impacts from TC and RF in Vietnam. We find that whether recovery takes 1, 3, or 5 years does not significantly affect the total impacts in each year. However, these recovery assumptions become crucial when we examine the proportion of assets damaged at any specific moment. For instance, a one-year recovery period would quickly reduce the percentage of impaired assets, while a five-year recovery would mean a part of the assets remains dysfunctional for a longer duration. This may in turn intensify secondary associated economic and social impacts^23^ such as business-interruption or the provision of basic services. This finding underscores the importance of tailoring the recovery model to the question of interest, which may also greatly differ on the hazards and the geographic locations or the communities affected^24–26^. Our framework is designed to accommodate this need, allowing users to integrate specific recovery timelines. These could also be on more detailed temporal scales than annual recovery estimates, provided that the hazard event dates are given on a higher temporal resolution. Furthermore, we here only considerd changes in exposures in-between events due to ongoing recovery, but the framework would also allow for an integration of changes in the vulnerability if reasonable parametric assumptions can be made.

Beyond the inclusion of recovery and common drivers, our framework allows studying the risk of multi-hazards consistently over custom spatio-temporal scales. For instance, we investigate the impact of either TC, RF or both at each exposure location on a $150''$ resolution globally in a given year. We also investigad the impacts of all pairings of TC, RF, and HS at each exposure location for Vietnam. Hence, one can study individual years, which can be useful in the development of storylines^27^, for instance by selecting an extreme compounding year with particularly many exposures impacted by both RF and TC globally, or by RF, TC and HS in the Vietnam case study. Looking into a few of these high-impact events and determining where people are affected by specific hazard combinations allows to develop these storylines, thinking critically about the associated impacts and potential adaptation measures that could be beneficial in a multi-hazard setting. In the global analysis, we further show the average annual risk of either TC, RF or both at each exposure point, which allows to identify regions that are particularly at multi-hazard (TC and RF) risk, as well as regions that are at risk of either or.

The presented framework contains all the elements required to perform a complete multi-risk assessment. With the example shown here, we focus mainly on the multi-hazard aspects and consider a simplified description of exposures and vulnerabilities with regard to the multi-risk framework. A more in-depth analysis should improve on these components. In our study, we use regionally calibrated impact functions for the assets, but only one global step function for the affected population due to a lack of better information available in the literature. This difference in vulnerability definition plays a central role in the results obtained. Additionally, our assumptions on recovery are simple and we only apply them to the exposure component of the risk. However, one should recognize that vulnerabilities may also be affected in the recovery process. For example, buildings or people that have been affected by a hazard event may be more vulnerable to a subsequent event (of the same hazard or of another) if they do not have sufficient time to recover^14^. In order to perform a full multi-risk assessment, exposure, and vulnerabilities must therefore both also be time-dependent^3^ to reflect the complexity of recovery. In most cases, the data availability and the complexity of the recovery process^25,28^ in a multi-hazard setting will probably hinder the use of hyper-detailed models, and thus modellers will have to choose the appropriate level of detail for their study. The same applies to hazard interactions, where other interactions than physical drivers could be incorporated. The here presented framework has the advantage of both accommodating simple component models and more complex ones, which makes it suitable for multi-risk analysis with incremental complexity.

While the presented results have a strong multi-hazard modelling approach, it is important to also acknowledge the limitation thereof. The estimates of TC impacts are based on the wind hazard as a proxy for all damages from TCs combined and do not account for the effects of TC flooding due to storm surge and torrential rainfall^20^. While we argue that considering wind as a proxy for all damages is a good approximation for a global assessment to account for yearly compounding risks, for higher time or space resolutions, it would be beneficial to consider the compound risks due to these sub-hazards. These could for instance be integrated in this framework by defining the spatially compounding risks by hour or days rather than by years. Note that even for global risk assessment using the wind hazard as a proxy has limitations in particular in a changing climate as TC wind and flood sub-hazards are expected to change differently^29,30^. Regarding RF, our results are limited by the modelling performance of the ISIMIP2 flood modelling cascade. Flooded areas and impacts derived with a similar method, but with observational climate forcing have shown to reproduce satellite observed flooded areas with regionally varying accuracy, depending also on the choice of the global hydrological model and the climate forcing^31^. Sources of uncertainties associated with the translation of the climate forcing into discharge refer to the exact timing and scaling of peak-runoff^32,33^ and the translation into spatially explicit flood-depth on the basis of Digital Elevation Models^34^. The exact representation of flooded areas may particularly depend on the adequate representation of flood protection levels. In this study, present flood protection standards are implemented coarsely by means of the FLOPROS^35^. Previous studies suggest that these protection standards alongside the continental depth-damage functions^36^ may lead to an underestimation of damage in high-income areas and an overestimation in low-income world regions^31^.

Further analysis should investigate other climate risks such as heatwaves, wildfires, and droughts, for which considering common physical drivers may be central as these can be strongly correlated. To this end, efforts such as ISIMIP providing consistent models across various hazards are crucial^37^. A limiting factor is that these models are not designed to generate large probabilistic event sets, which renders the study of tail risks challenging. For example the case of RF in this work, the number of years was limited to the number of combinations of GCM years and hydrological models. Additionally, only the maximum annual flood depth is provided with ISIMIP, limiting the event-based approach. The coverage of extreme events could be improved by modelling large samples of hydro-meteorological events consistent with the physics of the GCMs, for example using coupled statistical-dynamical models, as done here for TCs. These models can help us in the study of tail risks, while avoiding running expensive GCM simulations. For instance, the drivers considered here through the use of a statistical-dynamical TC model are the ENSO phase and the Global Mean Surface Temperature (GMST), two important factors to describe global climate conditions and which have been shown to modulate both TC and RF^38,39^. Further drivers could also be considered, depending on their relevance to the studied risks and spatio-temporal scale.

After evaluating the risk associated with two different hazards, we further illustrate how this could be done with three hazards for a case study in Vietnam. For spatially compounding risks, it will usually be necessary to consider the total aggregated risk due to all hazards. For spatio-temporally compounding risks, assessing pairs of risks is commonly done, but assessing risks resulting from three or more hazards may also be relevant^40^. Especially when assessing compounding risks occurring over a longer time period or at a larger scale, the presented framework can be extended to assess the risk more than two hazards occurring simultaneously.

In summary, our study represents a significant contribution towards a more holistic approach to risk modelling, emphasizing the significance of a multi-hazard perspective in uncovering the potential consequences of common physical drivers on climate risks. We provide a framework to assess the effects of climate change on multi-hazard risk on a relevant spatio-temporal scale, and to study individual events, which can also serve in the development of multi-hazard storylines. Our framework further opens the door for multi-risk modelling that considers the evolving dependencies among multiple hazards, recovery processes, and changing vulnerabilities over time, ultimately enabling more effective risk management strategies in the face of changing socio-economic and climate conditions.

## Methods

In the present section, we describe how both TC and RF are modeled at $1\;^{\circ }$C and $2\;^{\circ }$C, and how we calculate their risk considering exposure and vulnerability. We furthermore describe how we combine individually modeled impacts to assess the combined risk of TC and RF. We finally describe the HS hazard and the recovery models for the national case study.

### Tropical cyclone hazard

The TC hazard in CLIMADA consists of TC track sets, which are coupled with a parametric wind model to simulate a 2D wind field. In this work, we use the ISIMIP archive TC tracks generated by a statistical-dynamical TC model downscaled for four global climate models (GCMs) from the Coupled Model Intercomparison Project Phase 5 (CMIP5) archive (HadGEM2-ES, MIROC5, IPSL-CM5A-LR and GFDL-ESM2M) for RCP2.6 and RCP6.0 warming scenarios^41^. The parametric wind model implemented in CLIMADA computes the gridded 1-min sustained winds at 10 m above ground following Holland^42^. TC wind fields are modelled on a global spatial grid at $360'' \times 360''$ resolution based on Geiger et al.^43^. Geiger et al.^43^ additionally developed an emulator which derives a functional dependence between the simulated TC landfall time series of each GCM and global mean surface temperature (GMST) and ENSO. This emulator can be used to draw random samples from the entire TC event set (of all GCMs) to produce larger sets of TCs for each GCM, replicating the expected number of TC landfalls and mean landfall intensity in a given region and year considering ENSO and GMST (for details see Geiger et al.^43^). We emulate two sets of TCs, for two warming levels since pre-industrial time: $1\;^{\circ }$C and $2\;^{\circ }$C degrees of warming. We define the years corresponding to a warming level based on the 31 year running mean as reported by ISIMIP, considering 10 years above and below that level for each warming level^44^ (See SI Section 5.2 for more information on the binning of years). For each of the four GCMs and two warming levels, we emulate 25 samples, which coupled with the unique GCM years, results in approximately 2500 years of TC activity.

### River flood hazard

As for RF, we derive spatially explicit global maps of projected flooded areas and flood depth at a $150''$ resolution based on the CLIMADA implementation, including data of the ISIMIP2 derived output from the simulation rounds a^31^ and b^45^. The applied dataset includes the harmonized multi-model simulations of the six global gridded global hydrological models (GHMs) participating in ISIMIP2b for the scenarios RCP 2.6, RCP 6.0 and RCP 8.5. In this study, we only incude RCP2.6 and RCP6.0 to be coherent with the TC modelling. For those, the same GCMs as for the TC modelling procedure are available. For the hazard modelling, assumed are constant socio-economic conditions from 2005 regarding e.g., urbanisation patterns, river engineering and water withdrawal. The global annual flood maps were generated following the methodology previously applied in Willner et al.^46^: The runoff output of the GHMs is first harmonized with respect to the routing scheme using the river routing model CaMa-Flood (version 3.6.2) yielding daily river discharge at 15 arcmin resolution. For each grid-cell, we select the annual maximum daily discharge and fit a generalized extreme value distribution using L-moment estimators of the distribution parameters allowing for a model bias correction of each simulation GCM/GHM combination, following the approach by Hirabayashi et al.^47^. We map the return period of each event to the corresponding flood depth in a MATSIRO model run driven by observed climate forcings, which has been shown to be consistent with observation-based data^48^ providing flood depth at a 15 arcmin resolution. For the mapping, we take into account flood protection levels given in the “Merged layer” of the FLOPROS database^35^, applying a threshold procedure implying that, when the protection level is exceeded, the flood occurs as if there was no initial protection; below the threshold no flooding takes place. For the final assessment, we re-aggregate the high-resolution flood depth data from $0.3'$ to a $2.5'$ resolution ($5 \,\hbox {km} \times 5 \,\hbox {km}$) by retaining the maximum flood depth as well as the flooded area fraction, defined as the fraction of all underlying high-resolution grid cells where the flood depth was greater than zero. In this case, we define events as the yearly maximum flood depth at each grid point, for each year and GCM/GHM combination. We then consider the same warming levels as we did with TC, which results in approximately 600 unique years of global RF per level. This means the RF events are represented by a smaller sample than TC events.

### Exposures

We model population and physical asset stock values at a $150'' \times 150''$ resolution through the LitPop module in CLIMADA. This module provides a globally consistent methodology to disaggregate asset value data proportional to a combination of nightlight intensity and geographical population data^49^. In the case of assets, 26 countries and areas cannot be calculated to lack of data. For population, it makes use of the Gridded Population of the World datasets^50^. Both exposures are kept constant at 2018 values.

### Vulnerability

Vulnerability of assets to TC are modelled based on the regionally calibrated impact functions provided in CLIMADA^20^. In the case of RF, we use the global flood depth impact functions developed by the Joint Research Center of the European commission^36^. In order to estimate the population affected, a step function is defined. The threshold for RFs is set to a flood depth of 1m as done by Kam et al.^51^. In the case of TC, we set the threshold to 33/s or a category 1 hurricane as done by Geiger et al.^43^. For examples of vulnerability functions, see SI section 5.1.

### Impact calculation

Single hazard impacts are calculated by combining hazard, exposure and vulnerability, using the CLIMADA impact module^19^, resulting in 4 different impact models (for the population and assets exposures, as well as for the two hazards). In case of the RF impacts to assets, we normalize the expected annual impact based based on the EM-DAT database annual average impacts from 1980-2010 in 2018 inflation adjusted value (See SI section 5.3). This is done in order to obtain a realistic ratio between both hazards. But it should be noted that the observed impacts may be underestimating the total impacts, as impacts may not always be reported. In the EM-DAT database^15^, we filter for both disaster sub types “Tropical cyclone” and “Riverine flood”. We then compare these values to the ones for historical period impacts. In order to account for the growth in the total asset affected value, we normalize the historical observed impacts based on the global GDB of 2018. The ratio of historical average annual impact over the modelled one is then multiplied with historical and future impacts.

For TC, we first calculate impacts on an event basis, with each event corresponding to a track. For both TC and RF, each event has a tag referring to the year and the GCM driving that event.

### Case study on national level: Heat stress modelling

In the case study, we use the global data for TC and RF, which we select only for the country boundaries of Vietnam. In addition, we model people exposed to extreme heat stress. To do so, we calculate the mean daily environmental stress index (ESI), which is an approximation for the wet bulb globe temperature (WBGT) considering temperature, relative humidity and solar radiation^52^. This approximation has been shown to perform well in Vietnam compared to the explicit calculation for WBGT^52^. The International Labor Organisation defines that workers are at moderate health risk when exposed to WBGT exceeding $27\;^{\circ }$C, while they start facing a high risk when exposed to WBGT above $33\;^{\circ }$C^21^. We here define that people are affected by extreme heat if the daily average WBGT exceeds $33\;^{\circ }$C. Finally, as we are here considering events as being years we assume the people are exposed to extreme heat stress if this threshold is exceeded on a single day within a calendar year.

### Case study on national level: Recovery assumption

In the Vietnam case study, we pick one set of consecutive years from the same model which leads to important damages from both TC and RF (HadGEM2-ES RCP2.6 2006–2018). We then test how changing the assumption on the number of years before recovery affects the result. Here we only consider the effect of recovery on the exposures and keep the vulnerability constant. As a guiding principle, we assume that the part of an asset that is damaged cannot be damaged again. For a recovery time of $R>1$ years, the impact $I_{y}$ in year *y* defined in Eq. (1) is adjusted by considering the effect of the impacts from the previous $R-1$ years that have not yet recovered. Note that for clarity of reading, we shall omit in the notation all other variable sub/super-scripts from Eq. (1).

We define the total affected (or damaged) exposure value in year *y* due to impacts and recoveries from previous years as $A^y$. Assuming a constant rate of recovery over *R* years, the affected exposures in year *y* due to affected exposures in $y-1$ is reduced by 1/*R* and reads

5 $$\begin{aligned} {\tilde{A}}^{y-1}_k = \left( 1-\frac{1}{R}\right) A^{y-1}_k := \gamma A^{y-1}_k . \end{aligned}$$

For $R=1$, no affected exposure value is transferred to the next year since ${\tilde{A}}_k^y = 0$. The fully recovered part ${{\tilde{E}}}_k^y$ of the exposure value in a given year *y* is obtained by subtracting the total part that was affected in the previous year after applying the recovery rate,

6 $$\begin{aligned} {{\tilde{E}}}_k^y = E_k - {{\tilde{A}}}^{y-1}_k. \end{aligned}$$

The total affected exposure value in year *y* (shown in Fig. 7a) is then given by the value transferred from the previous year plus the impact on the recovered exposure only,

7 $$\begin{aligned} A^y_k&= {{\tilde{A}}}_k^{y-1} + {{\tilde{E}}}_k^y \cdot f_k^y \end{aligned}$$

8 $$\begin{aligned}&= \gamma A^{y-1}_k + (E_k - {{\tilde{A}}}^{y-1}_k) \cdot f^y_k \end{aligned}$$

9 $$\begin{aligned}&= \gamma A^{y-1}_k + I^y\left( 1- A^{y-1}_k \frac{\gamma }{E_k}\right) := \gamma A^{y-1}_k + I^y\Gamma _k^{y-1}, \end{aligned}$$

where $\Gamma _k^{y-1}$ is the recovery factor, $I^y$ is the impact and $f^y_k = I^y / E_k$ the impacted total exposure value fraction for year *y* for $R=1$ as defined in Eq. (1). In other words, the total value of affected assets in year *y* corresponds to the sum of the affected assets from the previous year that have not recovered $\gamma A^{y-1}_k$ and the total possible impact $I^y$ reduced by the recovery factor $\Gamma _k^{y-1}$. The new impact in year *y* excluding impacts from previous years (shown in Fig. 7b) is then given by

10 $$\begin{aligned} L^y_k&= {{\tilde{E}}}^y_k \cdot f^y_k \end{aligned}$$

11 $$\begin{aligned}&= (E_k - {{\tilde{A}}}^{y-1}_k)\cdot f^y_k \end{aligned}$$

12 $$\begin{aligned}&= I^y\left( 1 - A^{y-1} \frac{\gamma }{E_k}\right) = \gamma A^{y-1}_k + I^y\Gamma _k^{y-1} \end{aligned}$$

Thus, the new impact in a given year is smaller for larger $\gamma$, i.e., longer recovery times, as the recovery factor is smaller.

For spatially and spatio-temporally compounding defined in Eqs. (2)–(3), the impact $I^y_k$ must simply be replaced by the total $A^y_k$ or the new impact $L^y_k$. Note that for $A^y_k$ this would result in considering compounding of events in different years but for which the recovery times overlap, whereas for $L^y_k$ only events happening in the same year can compound. Finally, we remark that non-constant recovery rates could also be used by replacing $\gamma$ in Eq. (5).

## Supplementary Information

Supplementary Information.

## Data Availability

The TC track simulations are available for scientific purposes only and upon request from WindRiskTech (info@windrisktech.com). The ISIMP data to generate RF is available at https://zenodo.org/record/4627841#.Y_nyRy8w1hH. The population and asset data is available through the CLIMADA data API https://climada.ethz.ch/data-api/v2/docs. EM-DAT historical damages are available at https://public.emdat.be/. The HS hazard can be calculated based on the ESI formula provided by Kong et al.^52^, using the “hurs”, “tas”, and “rsds” variables from the ISIMIP2b data.

## Code availability

The simulations to quantify the TC impact were conducted using the Python tool CLIMADA, available at https://github.com/CLIMADA-project/climada_python/ (a frozen version was made available at 10.5281/zenodo.8383171). In particular, the tool to generate the artificial TC exposure time series is freely available at https://github.com/CLIMADA-project/climada_petals/blob/main/doc/tutorial/climada_hazard_emulator.ipynb. The scripts used in this work are available at: https://github.com/CLIMADAproject/climada_papers/tree/main/202403_multi_hazard_risk_assessment.
