# Supplementary material for: Global multi-hazard risk assessment in a changing climate
Source: Sci Rep. 2024 Mar 11;14:5875. doi: 10.1038/s41598-024-55775-2 (PMC11636830; doi:10.1038/s41598-024-55775-2)
Supplement: Supplementary file 1 — Supplementary Information. [file 41598_2024_55775_MOESM1_ESM.pdf]

Supplementary information to: **Global multi-hazard risk assessment under climate change**

Zélie Stalhandske[1,2*+], Carmen B. Steinmann[1,2+], Simona Meiler[1,2], Inga J. Sauer[1,3], Thomas Vogt[3], David N. Bresch[1,2], Chahan M. Kropf[1,2]

[1]Institute for Environmental Decisions, ETH Zurich, Zurich, 8092, Switzerland
[2]Federal Office of Meteorology and Climatology MeteoSwiss, Zurich-Airport, 8058, Switzerland
[3]Potsdam Institute for Climate Impact Research, Potsdam, 14473, Germany
[*]zelie.stalhandske@usys.ethz.ch
[+]These authors contributed equally to this work

1 Effect of climate change on single-hazard risks

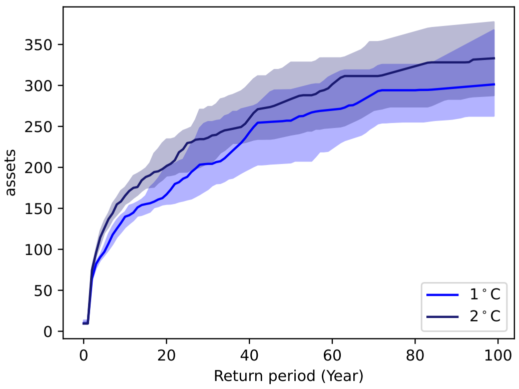

(a) Impact return period curves of RF on assets

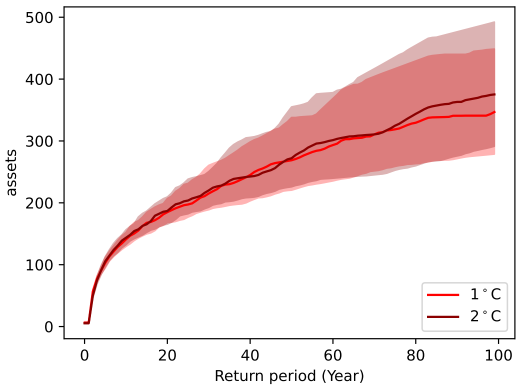

(b) Impact return period curves of TC on assets

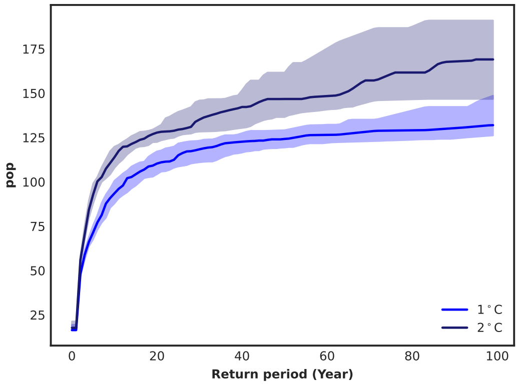

(c) Impact return period curves of RF on population

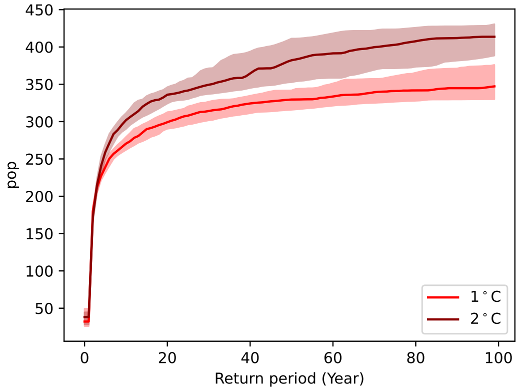

(d) Impact return period curves of TC on population

Figure S1. Impact return period curves of single hazard impacts at 1 °C and 2 °C of warming

2 Effect of common physical drivers on compound risks

For both levels of warming, the median curves of combined impacts appears to always be equal or higher when considering common physical drivers (see Suppl. Fig. S2).

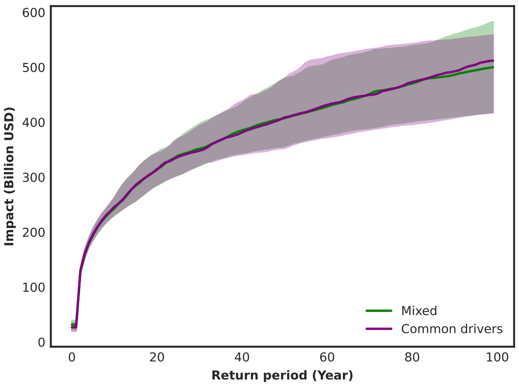

(a) Assets 1 °C

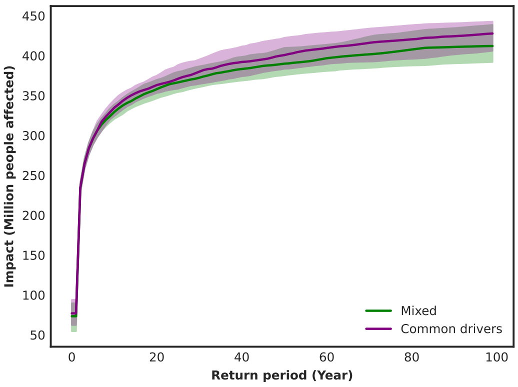

(b) Population 1 °C

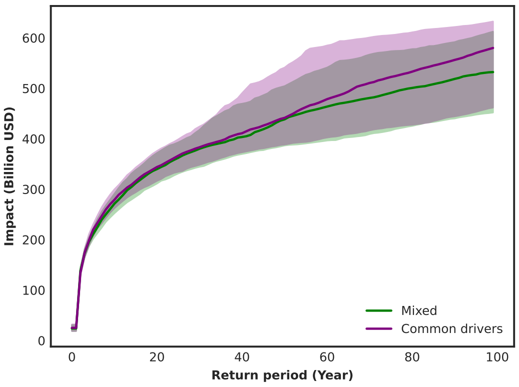

(c) Assets 2 °C

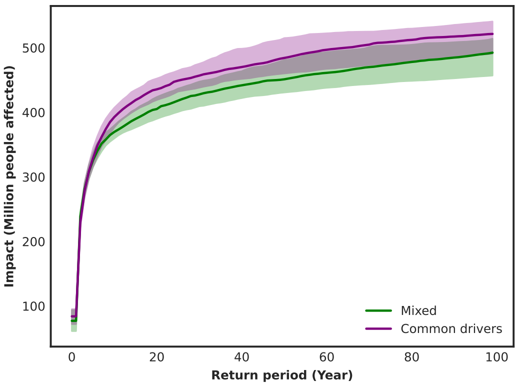

(d) Population 2 °C

Figure S2. Spatially compounding impact return period curves of combined impacts, when considering common physical drivers or randomly mixing years

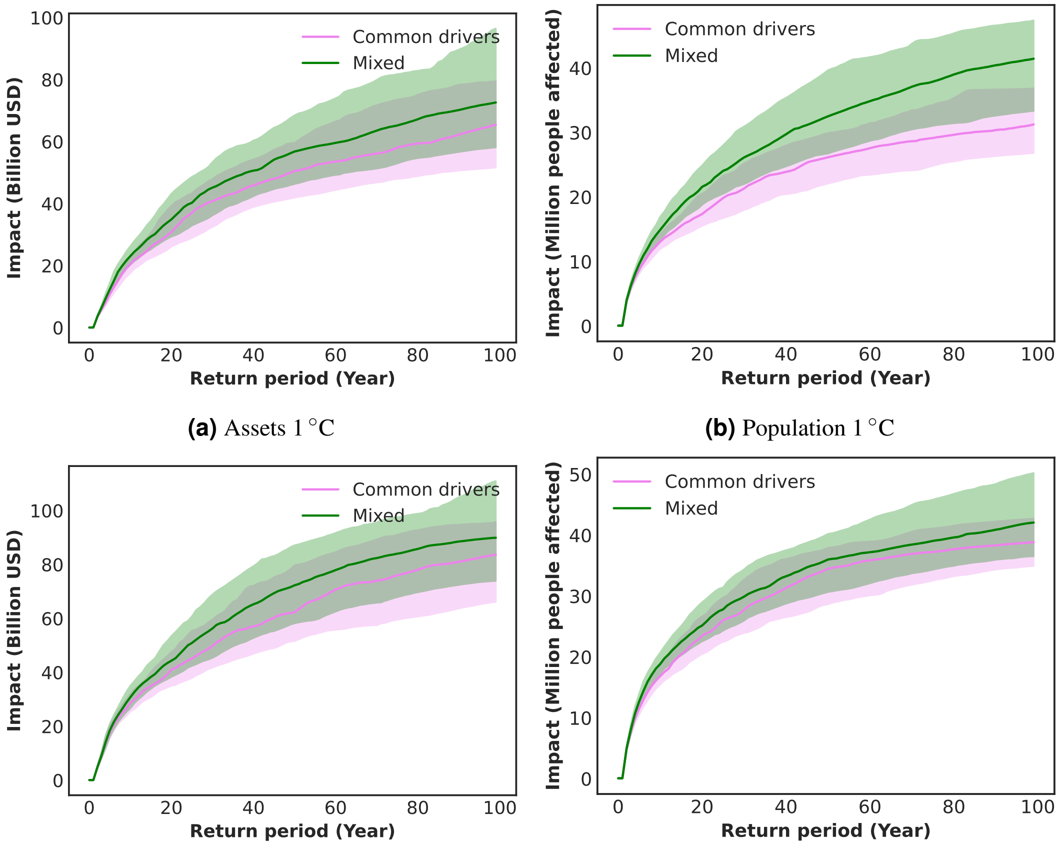

(a) Assets 1 °C

(b) Population 1 °C

(c) Assets 2 °C

(d) Population 2 °C

Figure S3. Spatio-temporal compounding impact return period curves at 1 °C and 2 °C when considering common physical drivers or randomly mixing years

3 Effect of climate change on spatio-temporally compounding impact return period curves

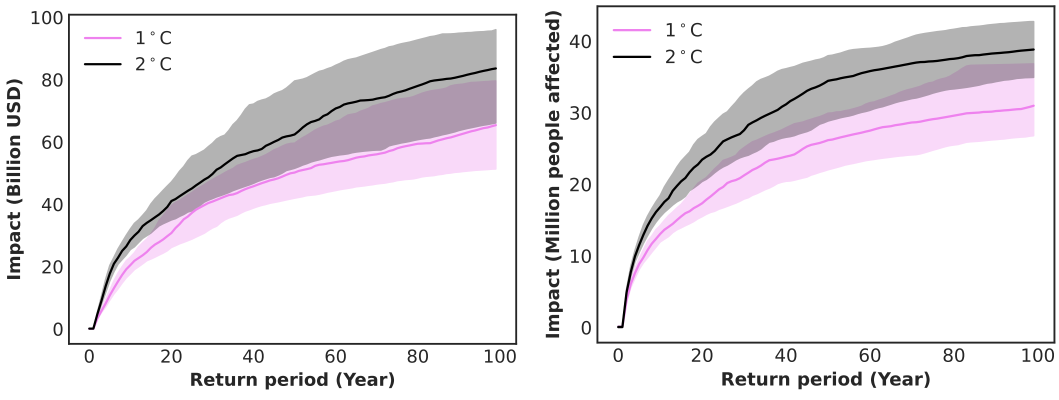

(a) assets

(b) population

Figure S4. Spatio-temporal compounding impact return period curves at 1 °C and 2 °C

4 Case study on national level

4.1 100-yr events

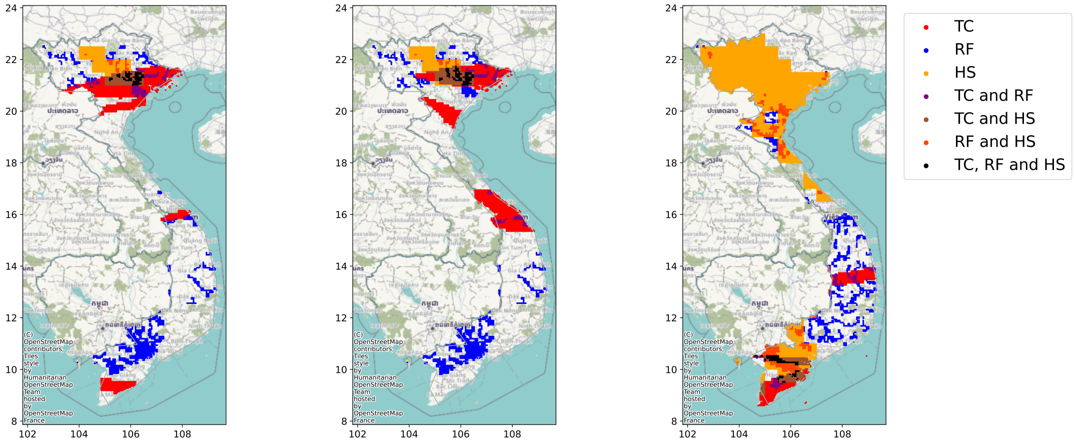

(a) Example events at 1 °C

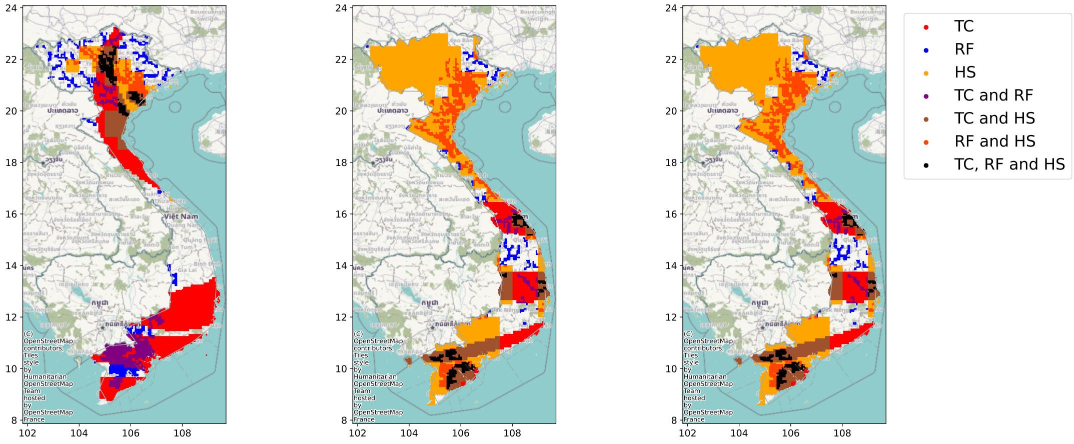

(b) Example events as 2 °C

Figure S5. Population exposure points affected by TC, RF and HS and their combinations in spatio-temporally compounding 100-yr events at 1 °C and 2 °C of global warming. The maps were generated using CLIMADA V4.0.1 (https://zenodo.org/records/8383171).

4.2 Effect of recovery assumptions on one exposure point

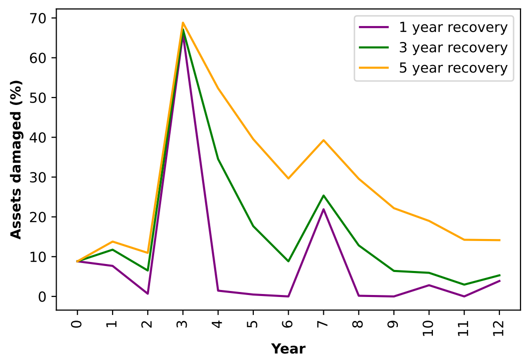

(a) Percentage of assets value damaged at one exposure point based on different recovery assumptions (1 year, 3 years and 5 years recovery) for combined TC and RF.

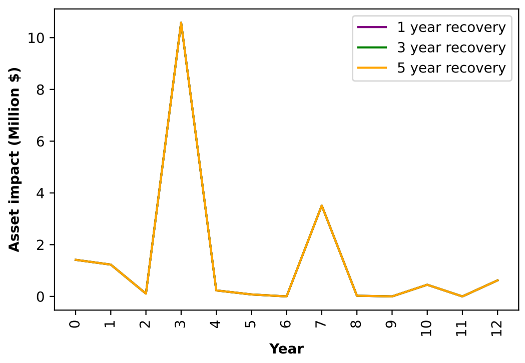

(b) Financial asset impacts at one exposure point based on different recovery assumptions (1 year, 3 years and 5 years recovery) for combined TC and RF.

Figure S6. Time series of assets value impacts in a given year for the HadGEM2-ES RCP2.6 2006-2018 for one exposure point

5 Supplementary methods

5.1 Vulnerabilities

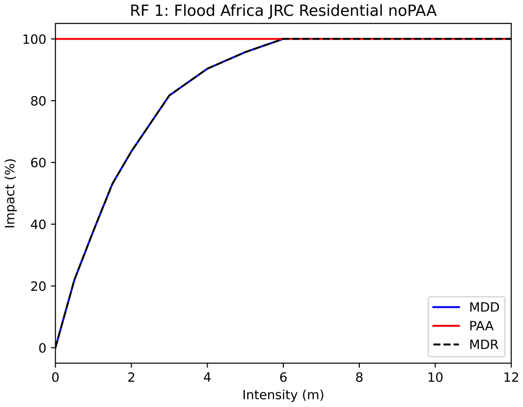

(a) RF Africa assets

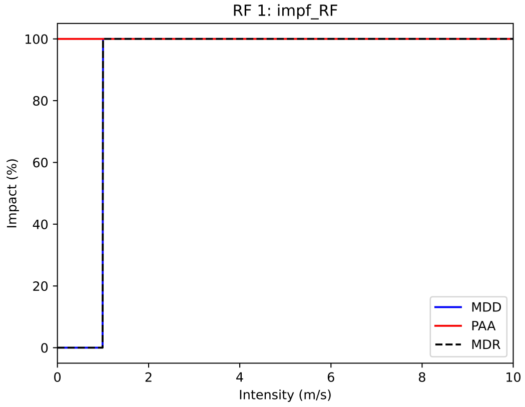

(b) RF population

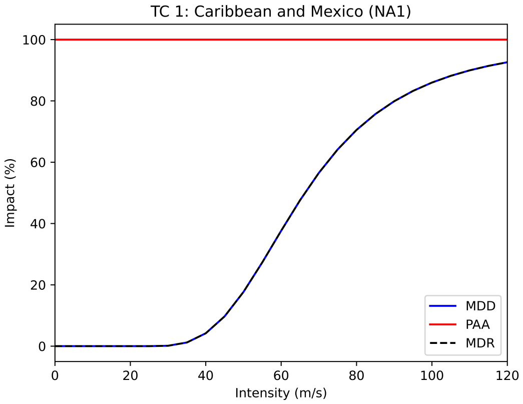

(c) TC Caribbean and Mexico assets

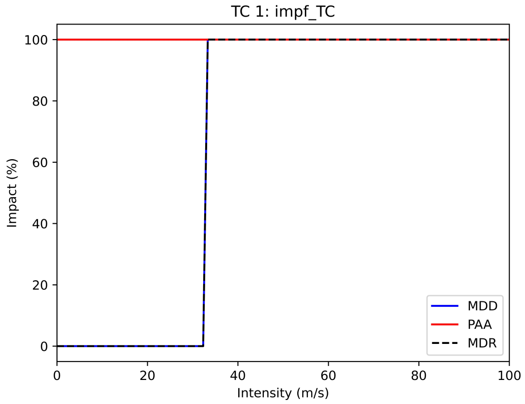

(d) TC population

Figure S7. Example of impact functions used in the impact calculation. In the case of assets, vulnerabilities are separated in 10 regions, while for RF there are a total of 6 regions. In the case of population, one function is used per hazard with a threshold.

In Fig. S7, examples of impact functions are shown. For assets, regional functions are defined based on Eberenz et al. (2021) for TC[1] and huizinga et al. (2017)[2] for RF. In the case of population, a step function is defined based on Kam et al. (2021) and Geiger et al. (2021)[3,4].

5.2 Warming levels

Table S1. Year bins for each GCM and RCP centered around the 31-year running mean of global mean tas reaching 1 °C or 2 °C based on ISIMIP provided values[5]. We consider +-10 years around the reported year as corresponding to that warming level. As the river flood data only starts in 2006, the full 21 years bin cannot be considered. The year bins were still taken as being centered around the warming level. This results in a total of 108 years for 1 °C, and 104 years for 2 °C.

		gfdl-esm2m	miroc5	hadgem2-es	ipsl-cm5a-lr
1 degree	RCP2.6	[2006, 2023]	[2006, 2025]	[2006, 2019]	None
	RCP6.0	[2006, 2027]	[2013, 2034]	[2006, 2023]	None
2 degree	RCP2.6	None	None	None	[2019, 2040]
	RCP6.0	[2066, 2087]	[2061, 2082]	[2040, 2061]	[2019, 2040]

5.3 Normalization of river flood impacts

In the case of river flood, we obtain $ 481 Billion average annual impact (AAI) using ISIMIP2a reanalysis data for the period 1980-2010 to calculate impacts on the LitPop asset exposure used in this work. Based on EM-DAT 1980-2010 values, historical reported impacts to be $ 48 Billion AAI, when adjusting for growth based on[6] and inflation adjusted to 2018 values. This is a factor 10 higher than the calculated impacts.

The large difference can be explained on the one hand by the fact that we use the maximal flood depth per grid point, which we combine with vulnerability curves developed at the building level, in order to calculate the impact. We expect this to lead to overestimates, but still allows to account for regional differences in vulnerability. Additionally, there is a wide spread in the impacts caused by each model combination. The study by Sauer et al. (2021) considered the median of the hydrological models at a regional level before summing impacts globally, additionally removing outliers[7]. Finally the EM-DAT database only contains observed events, while historical impacts may in reality be larger.

In the case of the present studies, the focus is not on the exact values of the average impacts, but rather on extreme values. We therefore give the same weight to all the model combination by considering the mean as the average annual impact. We however normalize the river floods impacts in order to have a realistic ratio between TC and RF impacts.

References

1. Eberenz, S., Lüthi, S. & Bresch, D. N. Regional tropical cyclone impact functions for globally consistent risk assessments. *Nat. Hazards Earth Syst. Sci.* **21**, 393–415, DOI: 10.5194/nhess-21-393-2021 (2021). Publisher: Copernicus GmbH.

2. Huizinga, J., Moel, H. d. & Szewczyk, W. Global flood depth-damage functions: Methodology and the database with guidelines. *JRC Res. Reports* (2017). Number: JRC105688 Publisher: Joint Research Centre (Seville site).

3. Kam, P. M. *et al.* Global warming and population change both heighten future risk of human displacement due to river floods. *Environ. Res. Lett.* **16**, 044026, DOI: 10.1088/1748-9326/abd26c (2021). Publisher: IOP Publishing.

4. Geiger, T., Gütschow, J., Bresch, D. N., Emanuel, K. & Frieler, K. Double benefit of limiting global warming for tropical cyclone exposure. *Nat. Clim. Chang.* **11**, 861–866, DOI: 10.1038/s41558-021-01157-9 (2021). Number: 10 Publisher: Nature Publishing Group.

5. ISIMIP. ISIMIP2b temperature thresholds and time slices (2017).

6. Worldbank. GDP growth (annual %) | Data (2023).

7. Sauer, I. J. *et al.* Climate signals in river flood damages emerge under sound regional disaggregation. *Nat. Commun.* **12**, 2128, DOI: 10.1038/s41467-021-22153-9 (2021).
